# A Characterization of Neurology Consults for Inpatients with SARS-CoV-2 Infection Compared to Other Respiratory Viruses

**DOI:** 10.3390/neurolint15040089

**Published:** 2023-11-23

**Authors:** Brian E. Emmert, Stephanie Gandelman, David Do, Kevin Donovan, Dennis L. Kolson, Matthew K. Schindler

**Affiliations:** 1 Department of Neurology, Perelman School of Medicine, University of Pennsylvania, Philadelphia, PA 19104, USA; 2 Department of Biostatistics, Epidemiology, and Informatics, Perelman School of Medicine, University of Pennsylvania, Philadelphia, PA 19104, USA

**Keywords:** neurologic sequelae of COVID-19, neurologic consultation and COVID-19, seizures and COVID-19

## Abstract

**Introduction:** Neurological consultation for patients infected with SARS-CoV-2 is common; it is currently unknown whether the neurologist’s approach to inpatient consultation of patients with SARS-CoV-2 should differ from the paradigm used to evaluate hospitalized patients with similar respiratory viruses. The goal of the present study is to determine if the preponderance of new neurologic diagnoses differs between inpatients with SARS-CoV-2 and similar non-SARS-CoV-2 respiratory viruses for whom neurology is consulted. **Methods:** We performed a retrospective chart analysis of inpatient neurologic consultations at three major Philadelphia-based hospitals. We compared the final neurologic diagnosis of 152 patients infected with SARS-CoV-2 to 54 patients with a similar ubiquitous non-SARS-CoV-2 respiratory virus (influenza A, influenza B, respiratory syncytial virus, rhinovirus, or adenovirus, the most commonly tested respiratory viruses at our institution). Secondary metrics included age, sex, level of care, prior neurologic diagnoses, and mortality. A multinomial logistic regression model was utilized to evaluate the relative difference between diagnostic category groups on all metrics. **Results:** The proportion of patients with seizure who were infected with SARS-CoV-2 admitted to an intensive care unit (ICU) was significantly higher than those who were admitted to a medical–surgical floor. SARS-CoV-2 was also associated with increased risk for ICU admission compared to other common respiratory viruses. SARS-CoV-2 inpatients requiring neurologic consultation were also more likely to be older and female as compared to the non-SARS-CoV-2 cohort. In other domains, the proportion of neurologic diagnoses between SAR-CoV-2 and non-SARS-CoV-2 respiratory viruses showed no significant difference. **Conclusion:** Patients requiring inpatient neurologic consultation with a diagnosis of SARS-CoV-2 infection or another respiratory virus were found to be remarkably similar in terms of their ultimate neurologic diagnosis, with the exception of a larger preponderance of seizure in critical-care-level patients with SARS-CoV-2 infection. Our study suggests that the neurological approach to patients hospitalized with SARS-CoV-2 should be similar to that for patients with similar common respiratory infections, noting that seizure was seen more frequently in critically ill patients infected with SARS-CoV-2.

## 1. Introduction 

When SARS-CoV-2 was first identified in 2019, and then became a daily presence on the inpatient hospital wards in the United States in early 2020, a preponderance of non-respiratory complications was noted in patients with acute SARS-CoV-2 infection [1], and particularly in hospitalized patients [2,3]. Early on, it became well known that infection with SARS-CoV-2 is associated with neurologic symptoms such as hyposmia, ageusia, headache, myalgias, and fatigue [4,5]. However, early reports identified neurologic symptoms in up to 30% of hospitalized patients with SARS-CoV-2 and in up to 80% of those with acute respiratory distress syndrome (ARDS) [4]. Further, early data suggested that SARS-CoV-2 was associated with more severe neurologic disorders, such as acute cerebral infarct, acute inflammatory demyelinating polyneuropathy (AIDP), encephalitis, and seizure, most commonly among those with severe respiratory disease [6,7,8,9,10]. Patients with SARS-CoV-2 infection who developed neurologic complications also had a significantly worse six-month functional outcome following discharge from the hospital as compared with those who did not have neurologic complications. However, the pathogenesis remained unclear [11].

Prior to SARS-CoV-2, six coronaviruses were known to be pathogenic in humans: HCoV-NL63, HCoV-229E, HCoV-HKU, HCoV-OC43, severe acute respiratory syndrome (SARS), and Middle Eastern respiratory syndrome (MERS) [12]. The mechanism of neurological pathophysiology of SARS-CoV-2 is thought to be similar to that of other coronaviruses pathogenic in humans [13]. While general cell entry is facilitated through ACE2 receptor binding, the mechanism for transneuronal spread is not clearly delineated, with proposed mechanisms including transmission via the lungs, hematogenous spread, or through the olfactory route [14]. Clinically, both SARS and MERS viruses have been associated with disease in both the peripheral and central nervous system, including critical illness neuromyopathy [15], acute demyelinating encephalomyelitis (ADEM), stroke, and Bickerstaff encephalitis [16,17,18]. The rate of neurologic complications associated with these viruses is small, ranging from 0.04–0.2% [15,16,17,18]. Similarly, experience with other respiratory virus pandemics, including the H1N1 influenza pandemics, has demonstrated that other respiratory viruses can have effects on the nervous system [19,20,21]. However, the early reports of serious neurological complications associated with SARS-CoV-2 led to the assumption that these disorders that arise from SARS-CoV-2 infection may differ from those due to other common respiratory viruses [22,23].

Neurologic sequelae in patients hospitalized with respiratory illnesses are not specific to SARS-CoV-2; rather, there are common known neurologic sequelae associated with other types of respiratory infections, including influenza A and B, adenovirus, and respiratory syncytial virus (RSV). These include, but are not limited to, febrile seizures, acute demyelinating encephalomyelitis, and acute inflammatory demyelinating polyneuropathy [24,25,26]. Influenza in particular has been reported to be associated with post-influenza encephalitis or encephalopathy (IAE), and though more common in pediatric cohorts, it has also been reported in adults [26]. Given the emerging reports of neurological complications in the context of hospitalized acute SARS-CoV-2 infection, we performed a retrospective analysis of hospital neurology consultations in patients who were SARS-CoV-2-positive and compared it to hospital neurology consultations in patients who were positive for other non-coronavirus respiratory infections. While coronaviruses, and by extension, SARS-CoV-2, have a predilection to have neurologic consequences, thus far, limited data exist comparing the acute neurologic sequelae of SARS-CoV-2 with other non-coronavirus respiratory viruses. The goal of the present study is to determine if the inpatient neurology consultant should approach the evaluation of neurologic phenomena in patients infected with SARS-CoV-2 differently from the way in which they approach those infected with common respiratory viruses. This information will be critical in guiding the inpatient consulting neurologist’s clinical decision making when evaluating patients with neurologic symptoms and concurrent SARS-CoV-2 infection.

## 2. Methods

This study was a retrospective chart analysis of people who had inpatient (hospital) neurologic consultations at the University of Pennsylvania Health System, a large, multicenter tertiary care academic medical center. This study was approved by the University of Pennsylvania Institutional Review Board. We identified two cohorts of patients: (1) patients infected with SARS-CoV-2 between the dates of 17 March 2020 (the date of the first case of SARS-CoV-2 with neurology consultation) and 30 June 2021 (the time of initial data procurement and analysis) and (2) a pre-pandemic cohort of patients infected with common non-SARS-CoV-2 respiratory viruses (including influenza A, influenza B, rhinovirus, respiratory syncytial virus, and adenovirus) between January 2019 and January 2020. This timing for the non-SARS-CoV-2 respiratory virus cohort, immediately prior to the time of the first case of SARS-CoV-2 case reported in the United States, was selected in an effort to minimize confounding by cases of co-infection, particularly given that coinfection testing was not yet universal in the spring and summer of 2020. In total, 152 patients with SARS-CoV-2 and 54 patients with non-SARS-CoV-2 respiratory viruses were identified during these respective periods. Inclusion criteria included patients who were diagnosed with SARS-CoV-2 or one of the non-SARS-CoV-2 respiratory viruses during the hospitalization in which neurology was consulted. Viral diagnosis was confirmed through PCR, either direct SARS-CoV-2/Influenza PCR testing or respiratory viral panel PCR testing. Patients who tested positive for SARS-CoV-2 or other non-SARS-CoV-2 respiratory viruses after the neurology consult date were excluded. Patients for whom a consult order was placed but the consult was canceled and thus not completed were also excluded from the analysis.

Basic demographic information, primary final neurologic diagnosis (the primary diagnosis conferred by the neurology consult team), secondary neurologic diagnoses (additional diagnoses conferred by the neurology consult team), acuity of patient (i.e., admitted to intensive care unit (ICU) or medical–surgical (non-ICU) floor), whether the consult was called as an acute stroke alert, whether the patient had any prior chronic neurologic diagnoses, and whether the patient survived to discharge were extracted from each chart.

The primary final neurologic diagnoses obtained were then categorized into the following groups: altered mental status (limited to toxic–metabolic encephalopathy and delirium); neuroinflammatory/infectious (including multiple sclerosis, neurosarcoidosis, acute inflammatory demyelinating polyneuropathy); acute neurovascular (infarct, hemorrhage, dural venous sinus thrombosis); acute peripheral neuropathy (critical illness neuromyopathy); chronic neurologic condition (specifically, neurologic conditions not related to acute infection, such as new diagnosis of a chronic neurologic condition and incidental chronic stroke, as well as recrudescence of a prior neurologic condition, such as multiple sclerosis pseudoflare); seizure; other (including sympathetic storm, new primary headache, and traumatic brain injury); and non-neurologic (including functional neurologic disorder, physiologic tremor, catatonia). The total number of patients who had a primary diagnosis in each category was tabulated for both the SARS-CoV-2 and non-SARS-CoV-2 respiratory virus groups. The rate of each diagnosis group was then calculated by dividing the total number of patients in that diagnostic category by the total number of people in the corresponding cohort, to account for the difference in population size of the two groups.

All statistical analyses were completed using R 4.1.1 [27]. First, summary statistics (mean and standard deviation for continuous, count and rate for categorical) for the following were computed for the respiratory virus group: subject age, sex, ICU status (yes or no), fatality, and prior neurological medical history (Table 1). Group difference testing for these variables between the diagnosis groups was carried out using a Welch two-sample *t*-test for continuous variables, a chi-square test for categorical variables with counts greater than five, and Fisher’s exact test for other categorical variables. Counts and rates for the categorized neurological diagnoses at visit were also computed for SARS-CoV-2 group and ICU status.

The rate of these various neurologic conditions as a function of ICU status and respiratory virus group was then modeled using a multinomial logistic regression model. Please see Appendix A for the statistical equation and explanation of analysis. This model was fitted to the data using the nnet R package [28]. While the log odds is modeled by default, estimates and confidence intervals (CIs) were instead computed for the rate of diagnosis for each neurologic condition, given their SARS-CoV-2 diagnosis and ICU status, using the inverse logit transformation. Rate in this case refers to proportion of those with a given neurological diagnosis, out of those in each SARS-CoV-2 diagnosis and ICU status group. Unlike the log odds, these rate estimates are invariant to the choice of “chronic neurologic condition” as the reference and serve as the main results for this analysis.

We considered two sets of hypothesis tests based on this model. First, within ICU status, we tested if the rate of each neurologic condition differed based on SARS-CoV-2 or non-SARS-CoV-2 respiratory virus diagnosis. Second, within SARS-CoV-2 diagnosis, we tested if the rate of each neurologic condition differed based on ICU or medical–surgical floor level of care. These were achieved by testing the corresponding linear contrasts for each neurologic condition based on the above model (a series of *t*-tests). We computed multiplicity-corrected *p*-values for each set of tests, using the standard Benjamini–Hochberg FDR correction method [29].

### 2.1. Standard Protocol Approvals, Registrations, and Patient Consent

This study was approved by the University of Pennsylvania Institutional Review Board, protocol number 850014. The IRB granted a waiver of consent based on the following criteria: the research protocol involves no more than minimal risk, the research could not be carried out practicably without the waiver, and the waiver will not adversely affect the rights or welfare of the subjects. Participant consent was not required and thus waived.

### 2.2. Data Availability

Data not provided in the article because of space limitations may be shared (anonymized) at the request of any qualified investigator for purposes of replicating procedures and results.

## 3. Results

Patients who were consulted on by the inpatient neurology team in the hospital setting and were infected with SARS-CoV-2 were significantly older (*p* = 0.005) and had a higher rate of being admitted to the ICU (*p* = 0.023) compared to those hospitalized with other respiratory infections (Table 1). In addition, there was a slightly higher proportion of females than males in the SARS-CoV-2 group than the non-SARS-CoV-2 group. However, this did not reach statistical significance. Analysis of the distribution of neurological diagnoses conferred by the neurology consulting team revealed that there was no association between respiratory infection diagnosis (SARS-CoV-2 vs. non-SARS-CoV-2) and neurologic condition diagnosis across the cohorts (*p* = 0.8). However, when analyzing within the SARS-CoV-2 infection cohort, the proportion of seizures for patients with acute SARS-CoV-2 infection in the ICU was significantly elevated compared to the proportion for patients with acute SARS-CoV-2 infection on the medical–surgical floors (*p* = 0.01).

These results indicated that patients with SARS-CoV-2 infection in the ICU had a significantly higher rate of seizure compared to patients infected with SARS-CoV-2 on the medical–surgical floors (floor CI 0–0.08, ICU CI 0.14–0.34, *p* = 0.01). Patients with SARS-CoV-2 infection in the ICU had a significantly higher rate of recrudescence of neurological symptoms attributed to chronic neurological diseases compared to those on the medical–surgical floor (*p* = 0.03, floor CI: 0.26–0.56, ICU CI: 0.01–0.37) (Table 2). This difference depending on level of care in cases of recrudescence was also noted among the non-SARS-CoV-2 respiratory virus group (*p* = 0.03 floor CI: 0.3–0.05, ICU CI: 0.11–0.29) (Table 2). No other significant differences were found for the rates for the remaining neurologic conditions when comparing respiratory virus diagnoses and acuity, including no higher proportion of acute neurovascular disease in the SARS-CoV-2 group (Table 2).

## 4. Discussion

The present study examines whether the distribution of neurologic disorders in patients with acute SARS-CoV-2 infection differs from that of a similar cohort of patients with other non-SARS-CoV-2 respiratory viruses. The purpose of this study was to answer the question of whether the discerning inpatient consulting neurologist should approach a patient with SARS-CoV-2 infection differently than they would other patients in similar circumstances. The present study demonstrated that, overall, there was no significant difference between the frequency of final neurologic diagnosis of patients consulted upon with SARS-CoV-2 infection as compared to those with non-SARS-CoV-2 respiratory virus infection. However, when further divided by acuity of the patient (i.e., intensive care (ICU) vs. non-ICU units), patients located in the ICU with SARS-CoV-2 infections were more likely to have seizures as compared to patients with SARS-CoV-2 infections on the medical–surgical floors.

The present study demonstrates that patients critically ill with acute SARS-CoV-2 infection have a higher incidence of seizure [30,31]. It is well documented that patients in the ICU are at a higher risk for seizures than the general hospitalized patient overall [32]. However, the present study demonstrates a new distinction in this finding for patients with SARS-CoV-2 infection—critically ill patients with SARS-CoV-2 infection had a higher rate of seizure compared to their counterparts with less severe illness, a finding that was not seen in patients critically ill with other respiratory viruses. Therefore, on the whole, we hypothesize that the clinical approach the neurologist takes while consulting on a patient with SARS-CoV-2 infection should not differ to that taken for similar patient populations. However, if a patient with SARS-CoV-2 infection is critically ill, clinical suspicion for seizure should be higher, especially when the consult question is for altered mental status.

Contrary to early reports from the beginning of the pandemic, the present study did not show a significant difference in the rate of acute infarcts in patients infected with SARS-CoV-2 as compared to those ill with other respiratory viruses [33,34,35]. While the observational nature of our present study limits our ability to explain the lack of dichotomy in the two groups, this study used a comparison group of other respiratory viruses rather than the general population, on which other studies have mostly focused [33,35]. Our study suggests that there may be other factors, such as systemic inflammation, that mediate this increased risk of acute stroke, and not solely SARS-CoV-2 infection, something that has been proposed for the finding of increased risk of acute infarct in influenza [36]. That being said, our study does contradict at least one prior retrospective study evaluating rate of acute infarction in SARS-CoV-2 as compared to influenza [23]. Given the conflicting results amongst multiple cohorts, further investigation will need to be completed to critically analyze this association.

There are several limitations to the present study. Given the retrospective chart review nature of this study, data were limited to only what was documented at the time of the encounter. Further, given the cohort design of the study, our study is not intended to examine neurologic findings in all patients with SARS-CoV-2 or determine a baseline frequency of each neurologic disease/diagnosis in patients who have infections with SARS-CoV-2 or other respiratory viruses. Rather, the goal was to examine just the subset of this population who are inpatients consulted on by neurology in an effort to guide clinical decision making for consultant neurologists; we feel that this study design completes this specific task. Thus, our results are limited in scope to inpatients who had neurology consults requested. Given the single-center design of our study, our data demonstrated wide confidence intervals, a consequence of the statistical underpowering. Further studies will require larger sample sizes, potentially in multiple centers. Finally, in both groups, there were several patients who passed away from virus-related causes prior to elucidating their final neurologic diagnosis, and thus only a suspected neurologic diagnosis was made.

As the pandemic progressed, a new pandemic emerged, more colloquially known as “long covid” but now commonly referred to as post-acute sequelae COVID-19 (PASC) [37]. PASC is defined by the United States Centers for Disease Control and Prevention (CDC) and the National Institutes of Health (NIH) as “signs, symptoms, and conditions that continue or develop after acute COVID-19 infection [38]”. Symptoms of PASC are not limited to the brain; almost every organ has been documented to be involved [39,40,41,42]. Regarding neurologic consequences of PASC, the most typical symptoms reported include “brain fog”, delirium, fatigue, sleep disturbances, and depression, with fatigue being the most commonly reported symptom [43]. This entity has been seen following all levels of severity of disease and seems to have little correlation with disease severity [37]. Notably, similar post-acute sequelae with neurologic involvement have not been described in other non-SARS-CoV-2 respiratory viruses, and this may potentially serve as another point of distinction in the neurologic evaluation of this patient population.

It would appear that PASC is specific to SARS-CoV-2; the same or similar entity has not been described for the other respiratory viruses examined in the present study. That being said, PASC has been likened to chronic fatigue syndrome (CFS)/myalgic encephalomyelitis (ME), as there are many overlapping symptoms, including fatigue, pain, psychiatric comorbidities, etc. [44] At present, there is a poor understanding of the pathophysiology of CFS/ME, but one prominent hypothesis is a chronic sequela of an influenza infection [45]. However, the relative constraint of CFS/ME to neurologic and psychiatric complaints, unlike PASC, which has more systemic, multi-organ involvement, starkly differentiates these two entities.

At the time of data collection for the present study, PASC had not been characterized, and the definition was evolving. As such, data on the presence of PASC were not collected. Of note, PASC can develop without hospitalization from SARS-CoV-2 infection. Future studies should consider the long-term neurologic sequelae of acute SARS-CoV-2 in defining the underlying prevalence and exact consequence of the virus for the nervous system. This will be an important distinction to make as the acute and chronic neurologic complications of SARS-CoV-2 may be starkly different and require different assessment and management. Additionally, there were few accepted treatments of acute SARS-CoV-2 during the time frame analyzed for this study. Treatment effects on neurological consequences from the acute infection were not able to be analyzed. As our treatments for acute infection evolved, vaccines were developed, and the virus mutated with different virulence, so the acute neurological consequences have also potentially evolved. Importantly, acute SARS-CoV-2 infection can still cause severe infection, particularly in older individuals, those who have not received vaccination, and those with other risk factors such as diabetes mellitus, preexisting heart and lung disease, and a suppressed immune status.

Following examination of the data, several future directions have been raised for continued areas of research. First, it is unclear whether SARS-CoV-2-positivity in ICU patients correlates with a new diagnosis of seizure or exacerbation of established epilepsy. Similarly, if it is associated with new-onset seizures, further evaluation is needed to determine the risk of seizure recurrence following recovery from SARS-CoV-2 infection. In a similar vein, our study does not determine the absolute or relative risk of seizure in critically ill patients infected with SARS-CoV-2 or other respiratory viruses. Further evaluation, with a different study design, such as a cohort study examining patients with respiratory viruses and with SARS-CoV-2 and calculating the rate of seizure in these populations, will better answer this question. The present study also raises the question of whether acute neurologic diagnosis in the inpatient setting correlates with long-term neurologic sequelae in the outpatient setting. Finally, it is necessary to delve deeper into the dichotomy between early reports of elevated risk of acute stroke in patients with SARS-CoV-2 and the lack of increased risk in our study when compared to a cohort of patients with non-SARS-CoV-2 respiratory viruses.

In summary, based on our analysis of patients who received neurological consultation while acutely infected by SARS-CoV-2 or other similar respiratory viruses on a medical–surgical floor, the inpatient neurologic consultant should take similar clinical approaches to both groups of patients. That being said, in the ICU, the frequency of seizures was higher in patients with SARS-CoV-2 compared to those with other respiratory viruses, potentially reflecting more severe disease and/or greater neurological involvement in the SARS-CoV-2 cohort; therefore, a lower threshold to evaluate and treat for seizure may be warranted. This study demonstrates that a nuanced approach to clinical reasoning while consulting on patients with acute SARS-CoV-2 infection should be taken, considering the patient’s illness severity, but exercising caution so as to not over-attribute neurologic conditions to the viral infection.

## Figures and Tables

**Table 1:** Demographic table for the present cohort, dichotomized by virus diagnosis, either SARS-CoV-2 or a non-SARS-CoV-2 respiratory virus (including influenza A, influenza B, respiratory syncytial virus, and adenovirus).

Characteristic	SARS-CoV-2, N = 152	Non-SARS-CoV-2, N = 54	*p*-Value ^1^
Age	61 (15)	53 (19)	0.005 *
Sex			0.3
Female	89 (59%)	27 (50%)	
Male	63 (41%)	27 (50%)	
ICU	66 (43%)	14 (26%)	0.023 *
Fatality	36 (24%)	17 (31%)	0.3
Neuro History	80 (53%)	27 (50%)	0.7
Diagnosis			0.8
Acute Peripheral Neuropathy	11 (7.2%)	3 (5.6%)	
Altered Mental Status	33 (22%)	10 (19%)	
Chronic Neurologic Condition	46 (30%)	17 (31%)	
Neuroinflammatory	3 (2.0%)	3 (5.6%)	
Neurovascular	21 (14%)	8 (15%)	
Non-neuro	18 (12%)	9 (17%)	
Other	4 (2.6%)	1 (1.9%)	
Seizure	16 (11%)	3 (5.6%)	

Mean (SD); n (%); ^1^ Welch two-sample *t*-test; Pearson’s chi-squared test; Fisher’s exact test. Abbreviations: ICU = intensive care unit. N = number of patients. SD = standard deviation. * indicates significant finding.

**Table 2:** Estimated neurological condition rates from multinomial logistic regression model conditional on SARS-CoV-2 diagnosis, compared between those who required ICU-level care and those who did not. For each neurologic condition and SARS-CoV-2 group, estimates and CIs for the rates among non-ICU and ICU patients are provided, along with the difference and *p*-value after correction for multiple comparisons. Significant differences in rates of recrudescence were found between those in and not in the ICU, for both non-SARS-CoV-2- and SARS-CoV-2-diagnosed patients. Significant differences in rates of seizure were found between those in and not in the ICU, but only among SARS-CoV-2 diagnosed patients (*p* < 0.05). * indicates significance.

Diagnosis	SARS-CoV-2 Diagnosis	Estimated Rate for Non-ICU (CI)	Estimated Rate for ICU (CI)	Rate Difference	*p*-Value
Altered Mental Status	non-SARS-CoV-2	0.18 (0.09, 0.32)	0.21 (0.07, 0.49)	−0.04	0.79
	SARS-CoV-2	0.2 (0.13, 0.3)	0.24 (0.15, 0.36)	−0.04	0.69
Neuroinflammatory	non-SARS-CoV-2	0.07 (0.02, 0.21)	0 (0, 1)	0.07	0.16
	SARS-CoV-2	0.03 (0.01, 0.1)	0 (0, 1)	0.03	0.16
Neurovascular	non-SARS-CoV-2	0.07 (0.02, 0.21)	0.36 (0.16, 0.62)	−0.28	0.16
	SARS-CoV-2	0.09 (0.05, 0.18)	0.2 (0.12, 0.31)	−0.10	0.16
Non-neuro	non-SARS-CoV-2	0.15 (0.07, 0.3)	0.21 (0.07, 0.49)	−0.06	0.75
	SARS-CoV-2	0.14 (0.08, 0.23)	0.09 (0.04, 0.19)	0.05	0.51
Other	non-SARS-CoV-2	0 (0, 0)	0.07 (0.01, 0.37)	−0.07	0.49
	SARS-CoV-2	0.02 (0.01, 0.09)	0.03 (0.01, 0.11)	−0.01	0.79
Peripheral	non-SARS-CoV-2	0.08 (0.02, 0.21)	0 (0, 0)	0.08	0.16
	SARS-CoV-2	0.1 (0.06, 0.19)	0.03 (0.01, 0.11)	0.07	0.16
Recrudescence	non-SARS-CoV-2	0.4 (0.26, 0.56)	0.07 (0.01, 0.37)	0.33	0.03 *
	SARS-CoV-2	0.4 (0.3, 0.5)	0.18 (0.11, 0.29)	0.21	0.03 *
Seizure	non-SARS-CoV-2	0.05 (0.01, 0.18)	0.07 (0.01, 0.37)	−0.02	0.79
	SARS-CoV-2	0.01 (0, 0.08)	0.23 (0.14, 0.34)	−0.22	0.01

## Data Availability

Any interested parties are welcome to reach out to authors to request deidentified data.

## Appendix A

Statistical model for logistic regression:

$$log\left(\frac{Pr[Neuro=k]}{\mathrm{Pr}\left[Neuro=reference\right]}\right)=\beta_{0k}+\beta_{1k}COVID+\beta_{2k}ICU+\beta_{3k}COVID*ICU$$

where k denotes a given neurologic condition, allowing the model parameters to vary by condition. Pr[*Neuro = k*] denotes the rate of neurological condition *k* given predictors, *reference* denotes a chosen reference condition, and *COVID* and *ICU* denote binary variables that equal 1 if the respective variables equal the stated category (SARS-CoV-2 diagnosis and ICU visit, respectively) and 0 otherwise. The parameters have the following interpretation: they denote the difference in the log odds of neurologic condition relative to chronic neurologic condition, between those diagnosed with SARS-CoV-2 and those with a non-SARS-CoV-2 respiratory virus, controlling for ICU. The other parameters follow similarly for their respective variables.
